# Who Listens, How We Share: Social Context Effects on Mood in Brief Reminiscence Among Older Adults in China

**DOI:** 10.1093/geroni/igaf122.3870

**Published:** 2025-12-31

**Authors:** Keyi Shi

**Affiliations:** Chinese University Hong Kong, St. Louis, Missouri, United States

## Abstract

Older adults face heightened risks of depression and affective dysregulation, particularly in low-resource contexts. Reminiscence, involving the structured recall of autobiographical memories, has been shown to improve emotional well-being, but most evidence comes from multi-session interventions in Western populations. The immediate effects of single-session reminiscence and the influence of social context, including listener familiarity and sharing modality, remain underexplored. I conducted a quasi-randomized, mixed-design study with 91 community-dwelling older adults (mean age = 67.7 years, 54% female) in Jiujiang, China. Participants were assigned to online writing, offline writing, oral sharing with a relative, or oral sharing with an unfamiliar young adult listener. Mood was assessed before and after the session using the Brief Introspection Scale (16 items; alpha = .84) and a global affect rating. A two-by-four mixed ANOVA tested main and interaction effects, followed by item-level analyses. No significant time by group interaction was observed (p > .05). Item-level analyses revealed significant pre-post increases in positive affective states including lively, content, loving, and active (all ps < .05), with trends favoring oral sharing over writing. Brief reminiscence improved specific positive affect and proved feasible in a low-resource, non-Western context. These findings highlight its potential as an accessible, person-centered strategy for enhancing emotional well-being and underscore the importance of social context in optimizing intervention benefits.

